# Effect of virtual reality on spatial–anatomical understanding in preoperative liver surgery: a randomized crossover study

**DOI:** 10.1038/s41598-026-61007-6

**Published:** 2026-07-08

**Authors:** Anton Zolkin, Christoph Rüger, Christopher Remde, Zeynep Akbal, Max M. Maurer, Nathanael Raschzok, Johann Pratschke, Igor M. Sauer, Moritz Queisner

**Affiliations:** 1 Department of Surgery, Experimental Surgery, Charité – Universitätsmedizin Berlin, Corporate Member of Freie Universität Berlin, Humboldt Universität zu Berlin, Campus Charité Mitte | Campus Virchow-Klinikum, Augustenburger Platz 1, 13353 Berlin, Germany; 2 BIH Charité Clinician Scientist Program, BIH Biomedical Innovation Academy, Berlin Institute of Health at Charité – Universitätsmedizin Berlin, Berlin, Germany; 3 Cluster of Excellence Matters of Activity, Image Space Material funded by the Deutsche Forschungsgemeinschaft (DFG, German Research Foundation) under Germany’s Excellence Strategy - EXC 2025 - 390648296, Berlin, Germany

**Keywords:** Anatomy, Health care, Medical research, Neuroscience

## Abstract

Precise spatial understanding of complex anatomy is critical for preoperative planning in hepatobiliary surgery. Traditional CT and MRI imaging require mental reconstruction of anatomy from 2D slices, imposing substantial cognitive load. Although 3D reconstructions improve spatial understanding, they are typically displayed on 2D screens, limiting true depth perception. Virtual Reality (VR) visualization offers both stereoscopic depth and embodied interaction to improve spatial-anatomical understanding, yet its quantitative advantage over standard desktop visualization remains uncertain, especially regarding task complexity. In this randomized crossover study, 58 medical students analyzed 3D liver models of varying complexity using both VR and desktop visualization. Performance on lesion/vessel relations and lesion segment allocation tasks served as a measure of spatial-anatomical understanding, while visuospatial ability was assessed with the Mental Rotations Test. In complex models, VR significantly improved performance compared with desktop visualization (28.0 ± 3.3 vs. 26.4 ± 3.6; *p* = 0.002, d = 0.46), whereas results for simpler models were comparable. The VR advantage scaled with task complexity and correlated with higher visuospatial ability (*r* = 0.31, *p* = 0.018). These findings indicate that VR is associated with measurable advantages under higher task complexity, supporting its potential role in surgical education and preoperative planning, although the present design cannot isolate which immersive features drive this benefit.

**Supplementary Information:**

The online version contains supplementary material available at 10.1038/s41598-026-61007-6.

## Introduction

 Spatial understanding of the human anatomy is a critical cognitive skill for every medical professional, especially surgeons, whose ability to internally visualize spatial relations during procedures is vital to safely and efficiently complete a procedure^1–5^. This skill is particularly important in hepatobiliary surgery, where the complex vascular and segmental anatomy of the liver demands high spatial awareness to achieve complete oncological resections while preserving sufficient functional liver tissue^6–8^.

Hepatocellular carcinoma (HCC) serves as a prime example of this challenge. For patients with HCC, surgical resection is the primary treatment option^9–11^. In the case of HCC, a full resection (R0) versus a resection with a microscopic residual tumor (R1) is one major factor that distinguishes between a long-term survival rate of 61.2% for R0 resections and 15.8% for R1 resections^12^. Hence, a failure to fully understand the spatial relationship between the tumor and surrounding vascular structures can lead to suboptimal resections, increasing the risk of recurrence and liver failure^13^. Therefore, accurate and intuitive preoperative planning is essential. The current standard of care in preoperative planning relies on two-dimensional (2D) imaging modalities, such as computed tomography (CT) and magnetic resonance imaging (MRI)^14^. Although sectional images provide high-resolution anatomical data, they require the surgeon to mentally reconstruct three-dimensional (3D) spatial relationships from 2D slices – a highly demanding cognitive task, particularly for trainees^15,16^. This mental burden can lead to reduced confidence and topographical uncertainty during surgery^17,18^.

3D reconstructions of medical imaging data have been available for over three decades and have demonstrated value in improving tumor localization and surgical decision-making^17,19–22^. Historically, the creation of patient-specific 3D models was both time-consuming and resource-intensive, limiting widespread adoption^23–25^. Recent advancements in computational power, particularly in artificial intelligence (AI) and machine learning, have significantly streamlined the creation of patient-specific 3D anatomical models through automated segmentation processes^26^. As a result, the segmentation time of a model was significantly reduced, making them more accessible and affordable, thereby enhancing their clinical availability and renewing interest in their use for preoperative planning^26–28^. Despite this progress, most of these 3D models are still displayed on conventional 2D desktop interfaces (DI), which lack stereoscopic depth cues and limit intuitive interaction. This constraint may reduce their effectiveness for spatially demanding surgical planning tasks. In this context, virtual reality (VR) provides users with a new way of assessing medical image data through the use of head-mounted displays, leveraging stereopsis and motion parallax to enhance depth perception and spatial cognition, alongside proprioceptive and vestibular feedback^29–32^. VR has shown promise in surgical education and planning by supporting more accurate spatial interpretation and faster anatomical orientation^13,18,33–35^. However, evidence of its effectiveness, particularly in complex surgical contexts, remains inconsistent.

While several studies suggest that VR enhances spatial understanding and decision-making compared to DI, the size and reliability of this effect vary considerably across studies^13,36,37^. For example, the comparative crossover study by Rashidian et al. demonstrated that VR significantly improved the accuracy of surgical decision-making in complex operative planning scenarios, whereas simpler anatomical exercises showed no difference between modalities^36^. This suggests that VR’s benefit may be most pronounced when cognitive demands are high. In contrast, other studies have not identified a significant advantage of VR^38^. Hattab et al. compared spatial understanding in VR and DI using both a liver model and a non-medical pyramid model. Their results showed no statistically significant performance differences between modalities^39^. A potential reason for these mixed findings is methodological variability. Additionally, many previous studies used outdated VR hardware, which lacked the resolution, tracking precision and responsiveness of current-generation systems. Moreover, few studies explicitly stratified task complexity^13,38,39^. Crucially, the question of whether VR provides incremental value primarily in complex tasks and whether this benefit diminishes for simpler spatial challenges has not been systematically studied.

Our study aims to address these gaps by evaluating spatial-anatomical recall of liver structures in VR versus DI modalities across different levels of complexity. Unlike prior studies, our research isolates modality effects by minimizing potential confounders through controlled participant selection, standardized hardware and consistent visual presentation. Participants’ stereoscopic vision, spatial reasoning ability and confidence are also assessed to explore individual factors that may influence modality effectiveness. We hypothesize that (1) H1: VR will lead to higher task performance scores in complex cases compared to DI-based visualization. (2) H2: The performance gap between VR and DI will be smaller in simpler cases, suggesting that VR’s benefits scale with task complexity. The overarching aim is to validate whether VR can meaningfully enhance spatial-anatomical understanding in preoperative planning, especially when surgical complexity demands more than conventional visualization can offer.

## Methods

### Study design

This study employed a randomized, comparative crossover design to evaluate the impact of visualization modality, VR versus DI, on spatial-anatomical understanding in preoperative planning. Participants assessed 3D liver models of varying complexity in both modalities across a single session. This within-subject design controlled for inter-individual variability and allowed comparison across complexity levels.

### Participants & ethics consideration

A total of 62 medical students from Charité – Universitätsmedizin Berlin were recruited. Eligibility criteria required participants to have completed at least their second year of medical studies to ensure a comparable baseline of anatomical knowledge, particularly of hepatic structures. Furthermore, this cohort was selected to minimize variability due to prior surgical experience, as participants had limited exposure to liver surgery. Exclusion criteria included uncorrectable visual impairments, motion sickness or epilepsy, all of which were assessed via a participant information sheet. Participants provided informed consent prior to their inclusion in the study. The study protocols and procedures were reviewed and approved by the Ethical Review Board of the Charité – Universitätsmedizin Berlin, Germany (EA2/132/24), and all methods were conducted in accordance with these guidelines and regulations. All collected data were anonymized and securely stored using in-house infrastructure to ensure compliance with clinical data protection standards.

### Study protocol

**Fig. 1:**
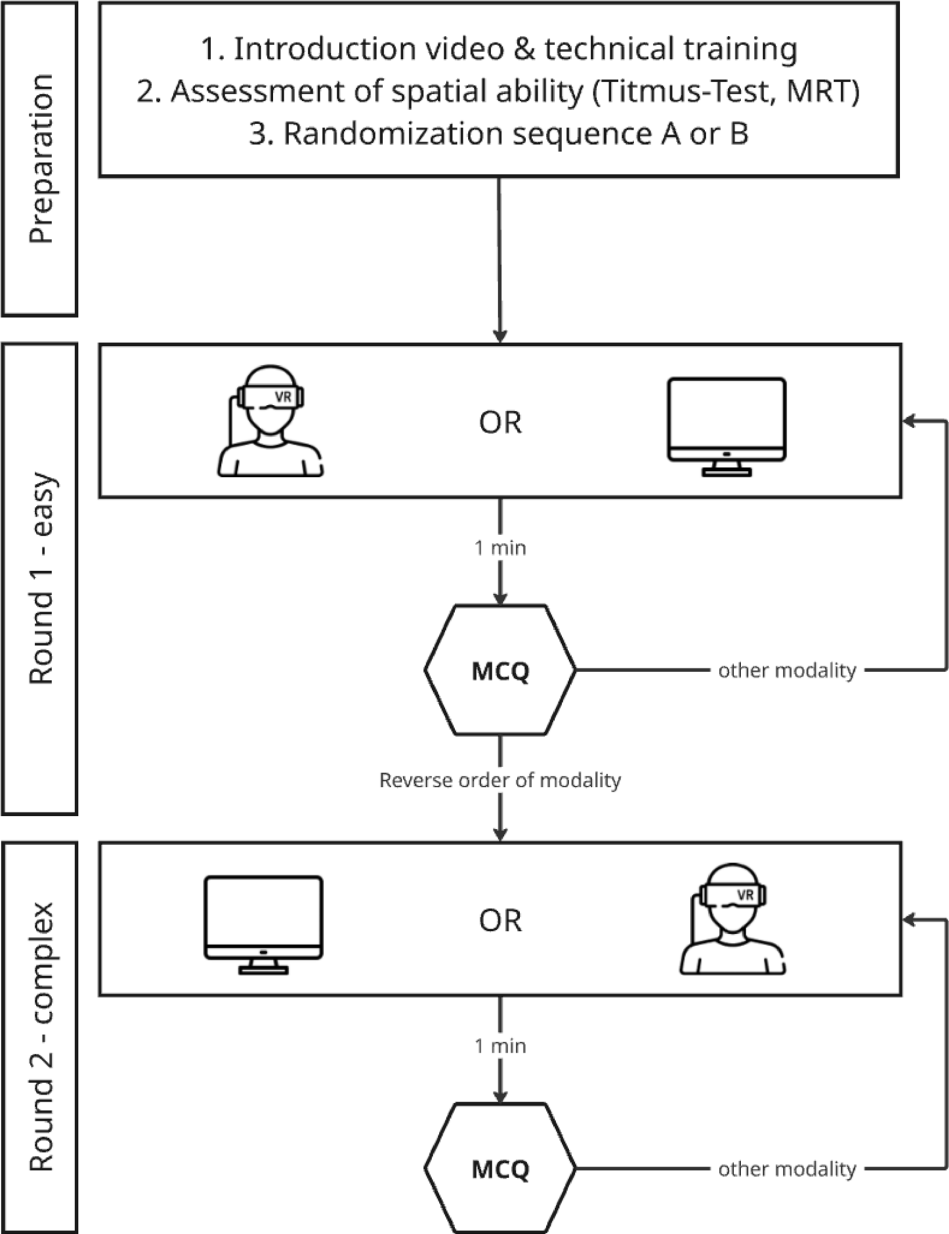
Study flow diagram. Flow of participants through preparation, randomization, evaluation of easy and complex liver models. Randomization sequence A followed the modality order VR-DI-DI-VR, while sequence B followed DI-VR-VR-DI. MRT mental rotations test; VR virtual reality; DI desktop interface; MCQ multiple-choice questionnaire.

Participants attended a single study session. Prior to the session, they viewed an 8-minute instructional video detailing liver anatomy, study objectives and questionnaire guidelines. Upon arrival, participants underwent a technical orientation, interacting with a training liver model in both VR and DI environments. This familiarization included two practice questions to ensure comprehension of the tasks and interface controls. Only participants who successfully completed this training proceeded to the study. Two standardized spatial ability assessments were administered before the experiment: the Titmus Stereotest to evaluate stereoscopic vision and the Mental Rotations Test (MRT) to assess visuospatial skills^40,41^. Participants were then manually randomized using a predefined sequence to one of two groups. Group A began with one case in VR, followed by two cases on DI and concluded with a final case in VR. Group B followed the reverse order. The first two cases presented a single lesion with low complexity, while the latter two contained two lesions with high complexity. For each model, participants could explore the anatomy for up to five minutes, followed by a one-minute retention break and subsequent questionnaire completion. This crossover structure ensured balanced exposure to both modalities across difficulty levels, while breaks between tasks prevented reliance on short-term memory and promoted engagement with long-term memory^29^. The workflow of the procedure is illustrated in Fig. 1.

### Materials

#### 3D liver models

Four high-resolution triphasic CT scans from patients who had undergone abdominal surgery were retrospectively selected. Cases were chosen to represent anatomical variation in hepatic vasculature and to mitigate learning effects during the experiment. Three-dimensional reconstructions were created using Ziostation REVORAS™, version 5.2.0 (Ziosoft, Newark, CA, USA). Segmentation included the hepatic parenchyma, arterial and venous systems, portal veins and tumors. An additional model was created for training purposes. All models were reviewed for anatomical and clinical accuracy by two board-certified surgeons. Final meshes were exported in Wavefront Object File format (.obj) for software integration.

**Fig. 2:**
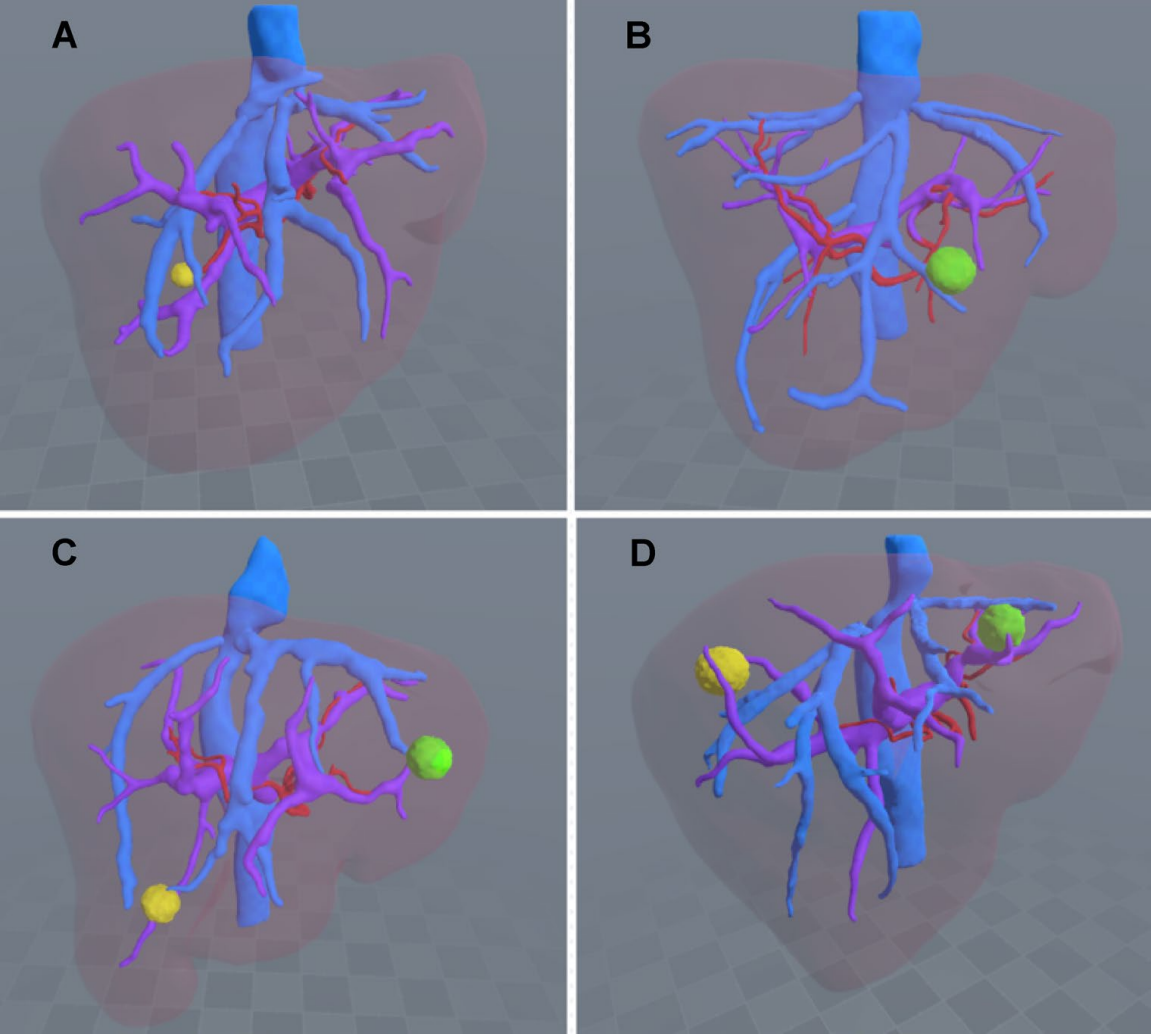
Liver models used for easy and complex tasks. Four three-dimensional liver models used in the experiment are shown. (**A**, **B**) Models used for easy task; (**C**,** D**) models used for complex task.

Lesions were artificially inserted into the models to standardize difficulty levels across cases. Two models contained a single lesion and two models contained two lesions (Fig. 2). The segmentation was simplified to include only the segmental branches of the liver vessels to enhance usability and ensure clear reference points for the questionnaire, while retaining clinical relevance (Fig. 2). Task complexity was therefore operationally defined as the number of lesions per model, one in the easy condition and two in the complex condition, while anatomical variation in vascular morphology was present across cases and balanced across complexity levels. No quantitative anatomical complexity metric (e.g., lesion–vessel distance, segment-boundary involvement, vessel-branching depth) was applied. Each additional lesion adds further lesion–vessel relationships that must be localized and tracked at the same time, which was expected to raise the cognitive load of the complex condition relative to the easy condition. Cognitive load was not measured directly in this study.

#### Software and hardware

A custom application, EXPLORE, was developed in-house using the game engine Unity, version 2022.3 (Unity Technologies, San Francisco, CA, USA) to visualize the 3D liver models in both VR and DI modalities with equivalent layout, lighting and model quality. The application operated at 60 frames per second in both environments without technical glitches.

**Fig. 3:**
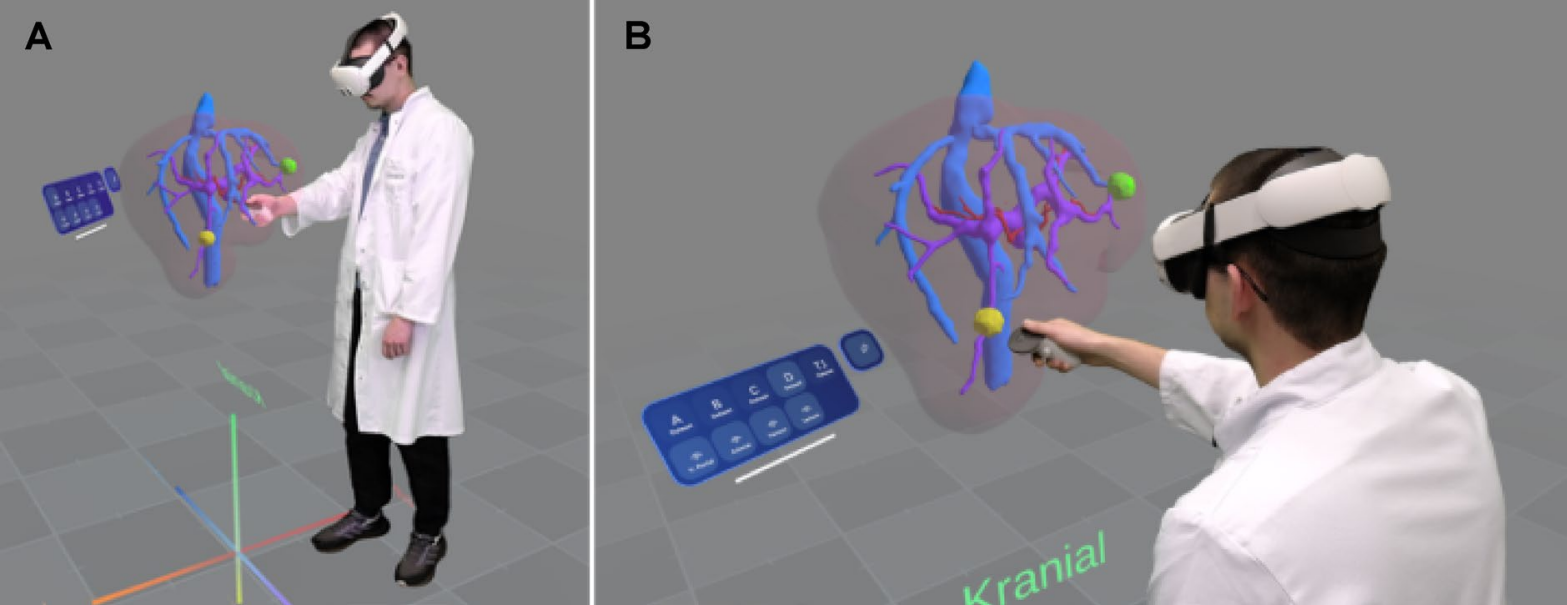
Experimental setup for the VR condition. Fully immersive virtual environment as viewed through the head-mounted display from two perspectives. (**A**) An anatomical orientation marker is positioned on the ground plane to indicate anatomical directions. (**B**) A complex liver model with two lesions is displayed centrally, together with an interactive blue control panel that allows users to switch between liver models and selectively filter vascular structures. Interaction within the VR condition is performed using a single handheld controller, shown in the user’s right hand.

In the VR condition, the application was deployed for Meta Quest 3 headsets (Meta Platforms, Menlo Park, CA, USA; Meta Horizon OS version 69; 2,064 × 2,208 pixels per eye; 72 Hz refresh rate). Participants interacted with the model using a single controller, enabling zooming, vertical (y-axis) adjustment and vessel system filtering. Model manipulation along the x- and z-axes was intentionally disabled to promote embodied spatial interaction. Thus, participants explored the model by physically moving around it, leaning closer to inspect details, or changing their viewing angle through natural head and body movement. This setup allowed the same functional operations as in the DI condition: scaling, rotation and viewpoint change, but achieved through physical navigation rather than input through mouse and keyboard (Fig. 3).

**Fig. 4:**
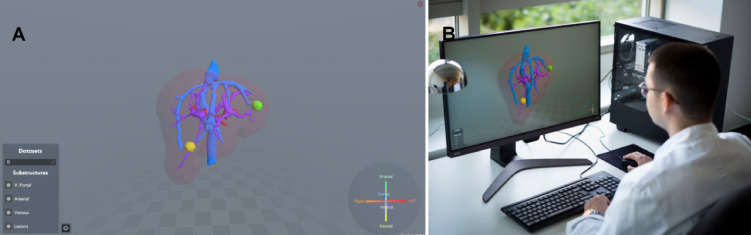
Experimental setup for the DI condition. (**A**) The user interface of the DI condition is shown. An anatomical orientation marker is displayed in the lower right corner, the control panel is positioned in the left corner and a complex liver model is shown centrally within the interface. (**B**) The interaction within the DI condition is performed using a computer mouse.

The DI version ran on a computer equipped with an AMD Ryzen 3950X CPU (Advanced Micro Devices, Santa Clara, CA, USA), Nvidia GeForce RTX 3090 GPU (NVIDIA Corporation, Santa Clara, CA, USA) and 64 GB RAM. The monitor resolution was 2560 × 1440 px at 60 Hz. Interaction relied on standard mouse and keyboard controls: scaling via scroll wheel, rotation and translation along all axes via click-and-drag. Vascular structures could be filtered identically to the VR condition. The user interface was matched across modalities to ensure functional equivalence, differing only in interaction mode, manipulation through mouse and keyboard in DI versus embodied navigation in VR (Fig. 4).

### Testing instruments and measures

The primary outcome measure was a spatial understanding questionnaire adapted from Hattab et al.^39^. Each model assessment included six questions (Supplementary Data 1). The first four questions evaluated the lesion’s spatial relationship to hepatic vessels (medial/lateral, cranial/caudal, dorsal/ventral and vessel contact yes/no), with one point awarded for each correct response (maximum of 4 points per question). The final two questions assessed the identification of the liver segment containing the lesion and the vascular pathway of a critical vessel system. Each was scored as either correct or incorrect, with two points for a fully correct response and none otherwise.

Participants also rated their confidence for each question on a 5-point Likert scale (1 = very unsure; 5 = very sure). Reference answers were established by two board-certified surgeons in the VR environment and reviewed by the study lead, with discrepancies resolved through discussion. To evaluate baseline spatial abilities, participants completed two standardized tests at the start of the session. Spatial ability was assessed using the revised Mental Rotations Test (MRT) by Peters et al., which requires participants to identify rotated versions of target figures^40^. A maximum of 24 points could be achieved, with points awarded only for fully correct responses. Stereoscopic vision was evaluated using the Titmus Stereotest, a widely used clinical tool to measure depth perception based on binocular disparity^41^.

### Statistical analysis

Statistical analyses were conducted in Python, version 3.10.12, using pandas, version 2.2.2, for data handling, Pingouin, version 0.5.4, for statistical testing and seaborn version 0.12.2, for visualization^42–44^. The significance level was set at α = 0.05, with the Benjamini–Hochberg method applied to control the false discovery rate across hypotheses. The primary hypothesis that performance on complex tasks would be higher in the VR condition than in the DI condition was tested using a one-sided paired t-test. Given the crossover design, all analyses were conducted within subjects and task scores were treated as continuous variables. A second paired t-test assessed whether the performance difference between VR and DI was larger for complex tasks than for easy tasks. To account for differing score ranges, complex-task MCQ scores were divided by two before comparison. The secondary hypothesis, examining whether spatial ability modulated VR-related performance differences, was evaluated using Pearson correlation between MRT scores and performance differences between modalities for complex tasks. Statistical significance was assessed using a two-sided test.

Confidence and calibration analyses were exploratory and were not included in the false-discovery-rate correction. Modality differences in confidence were tested with the Mann–Whitney U test, and the confidence–accuracy association was quantified per modality using the Goodman–Kruskal gamma coefficient, a rank-based measure that makes no assumption about the spacing of the Likert categories, with bootstrapped 95% confidence intervals (5,000 iterations).

## Results

### Demographics and participant characteristics

A total of 62 medical students were recruited for the study. Four participants did not complete the protocol and were excluded from analysis: two due to motion sickness, one due to incomplete questionnaire data and one due to difficulty understanding the medical terminology of the questionnaire. The final sample consisted of 58 participants (mean age = 25.10 ± 2.39 years), with an equal gender distribution (50% female, 50% male). All participants demonstrated normal stereoscopic vision as assessed by the Titmus test. The mean score on the MRT was 12.86 ± 4.83 for female participants and 12.69 ± 4.86 for male participants. With respect to prior experience, 81% of participants had never used VR before, while 19% reported occasional use. In contrast, 3D DI experience was more common: 16% had no prior exposure, 66% reported occasional use and 19% reported regular use. Surgical exposure within this cohort was limited: 67% had never observed a liver surgery, while 29% had observed or assisted in 1–5 and 3% in more than 10 procedures.

### Task performance

**Fig. 5:**
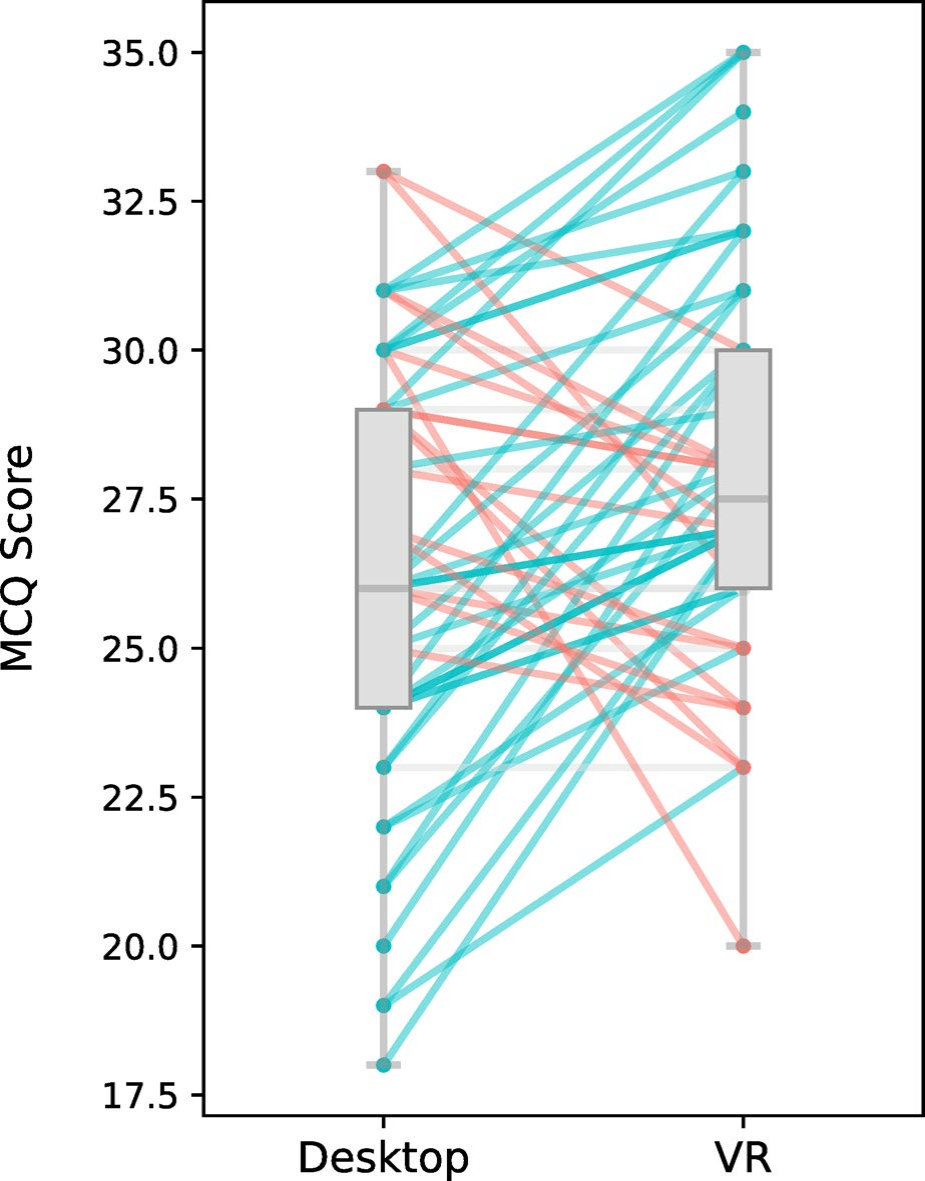
Comparison of performance on complex tasks between DI and VR conditions. Distribution of MCQ scores for the DI and VR conditions, shown as boxplots with paired participant data. Lines connect individual participants across conditions, indicating changes in performance between modalities. Improvements in MCQ score are shown in green (60.3%), no change in grey (12.1%) and declines in red (27.6%). VR virtual reality; MCQ multiple-choice questionnaire

Participants performed significantly better in the VR condition than in the DI condition for complex tasks. The mean score for VR was 28.00 ± 3.32 versus 26.40 ± 3.61 for DI, corresponding to a significant difference (*p* = 0.002) and a moderate effect size (Cohen’s d = 0.46) (Fig. 5).

**Fig. 6:**
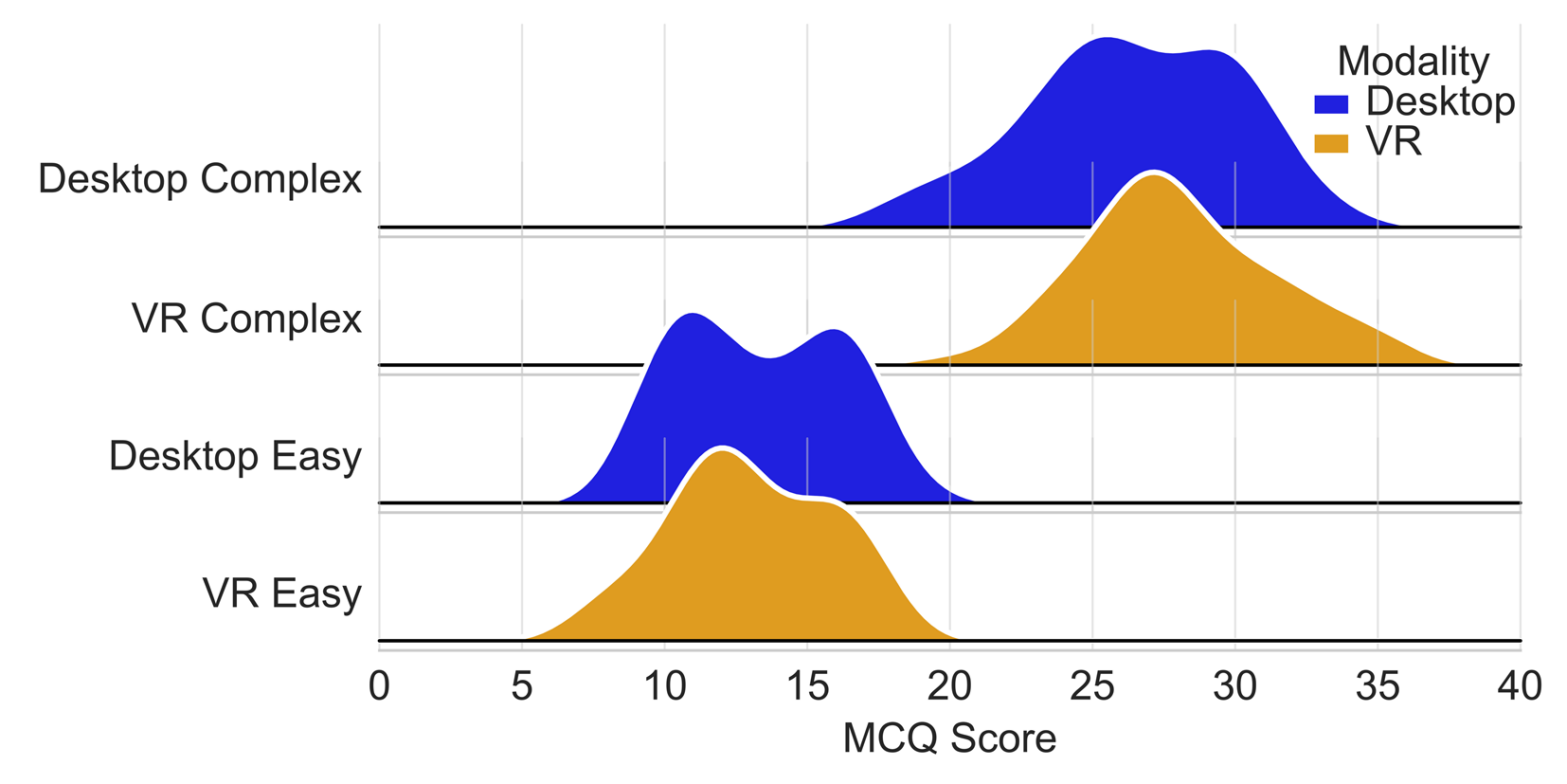
Distribution of task performance by modality and task complexity. Ridgeline plots show the distribution of MCQ scores for the DI and VR conditions across easy and complex tasks. Density curves represent the score distributions within each condition. VR virtual reality; MCQ multiple-choice questionnaire

The difference in performance between VR and DI was larger for complex tasks than for easy tasks. For complex tasks, the mean VR–DI difference was +1.60 ± 4.13 points, whereas for easy tasks it was −0.33 ± 4.60 points (p = 0.035), with a small-to-moderate effect size (Cohen’s d = 0.32) (Fig. 6).

**Fig. 7:**
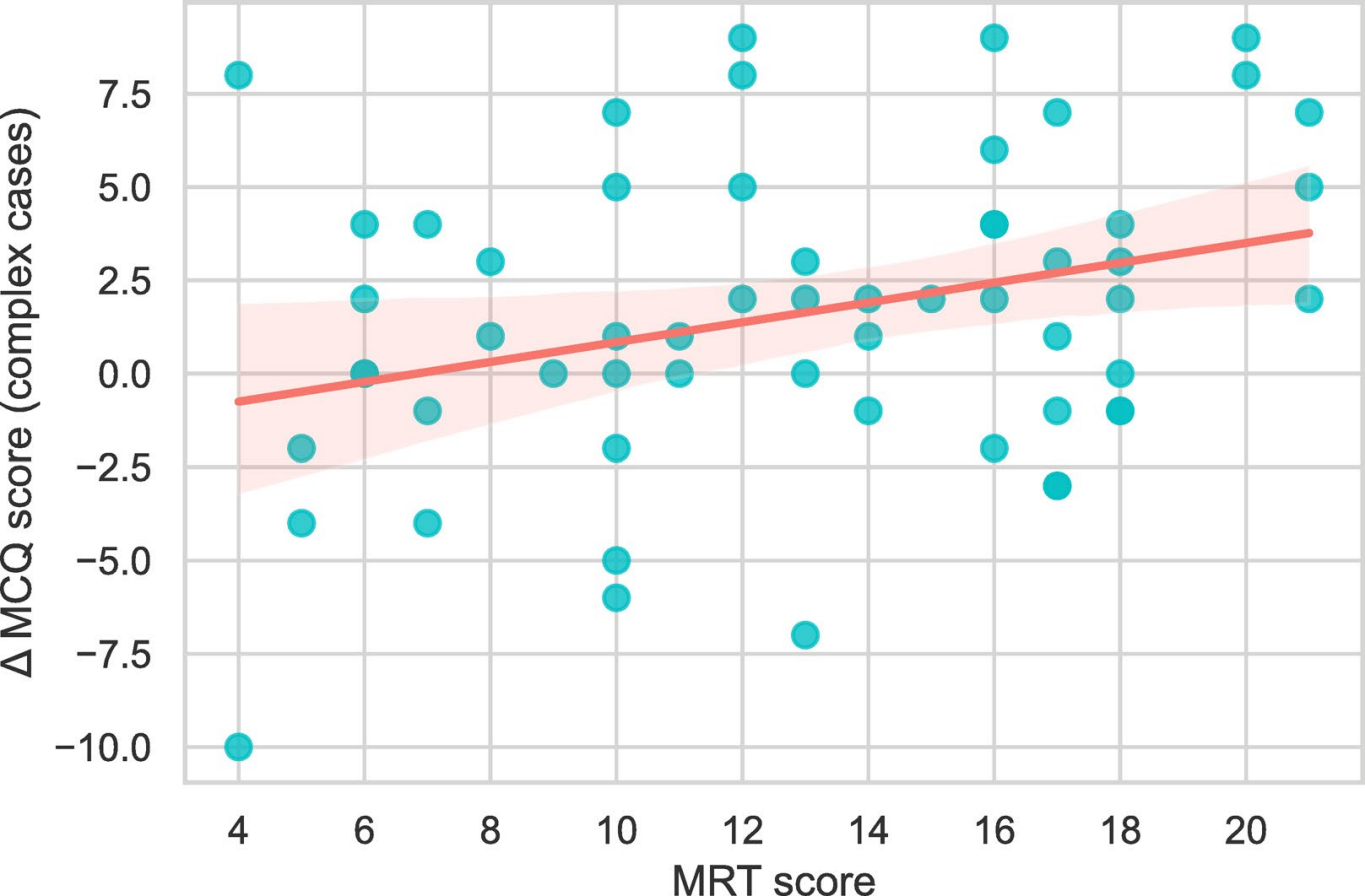
Association between spatial ability and performance differences between modalities for complex tasks. Scatter plot showing the relationship between MRT scores and the difference in MCQ scores between the VR and DI conditions for complex tasks. Each point represents an individual participant. The solid line indicates the linear regression fit, with the shaded area denoting the 95% confidence interval. MRT, mental rotations test; MCQ, multiple-choice questionnaire.

MRT scores were positively associated with modality-related performance differences for complex tasks (Pearson *r* = 0.31, *p* = 0.018), indicating greater VR-related benefits among participants with higher spatial ability (Fig. 7).

### Confidence

**Fig. 8:**
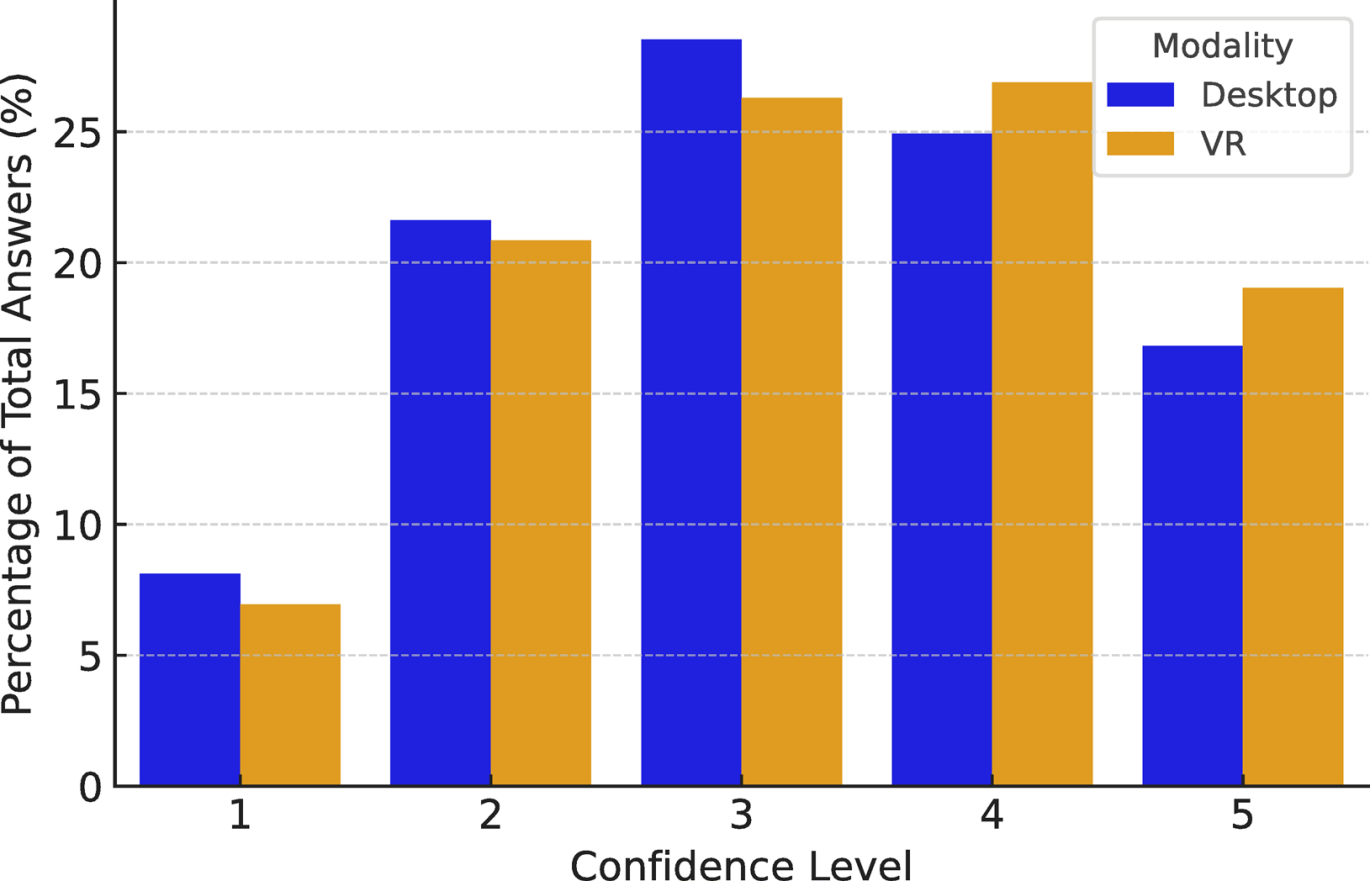
Distribution of self-reported confidence levels for the two binary-scored items by modality. Bar plots show the distribution of reported confidence levels for DI and VR conditions. Values represent the percentage of total responses within each modality. VR virtual reality.

**Fig. 9:**
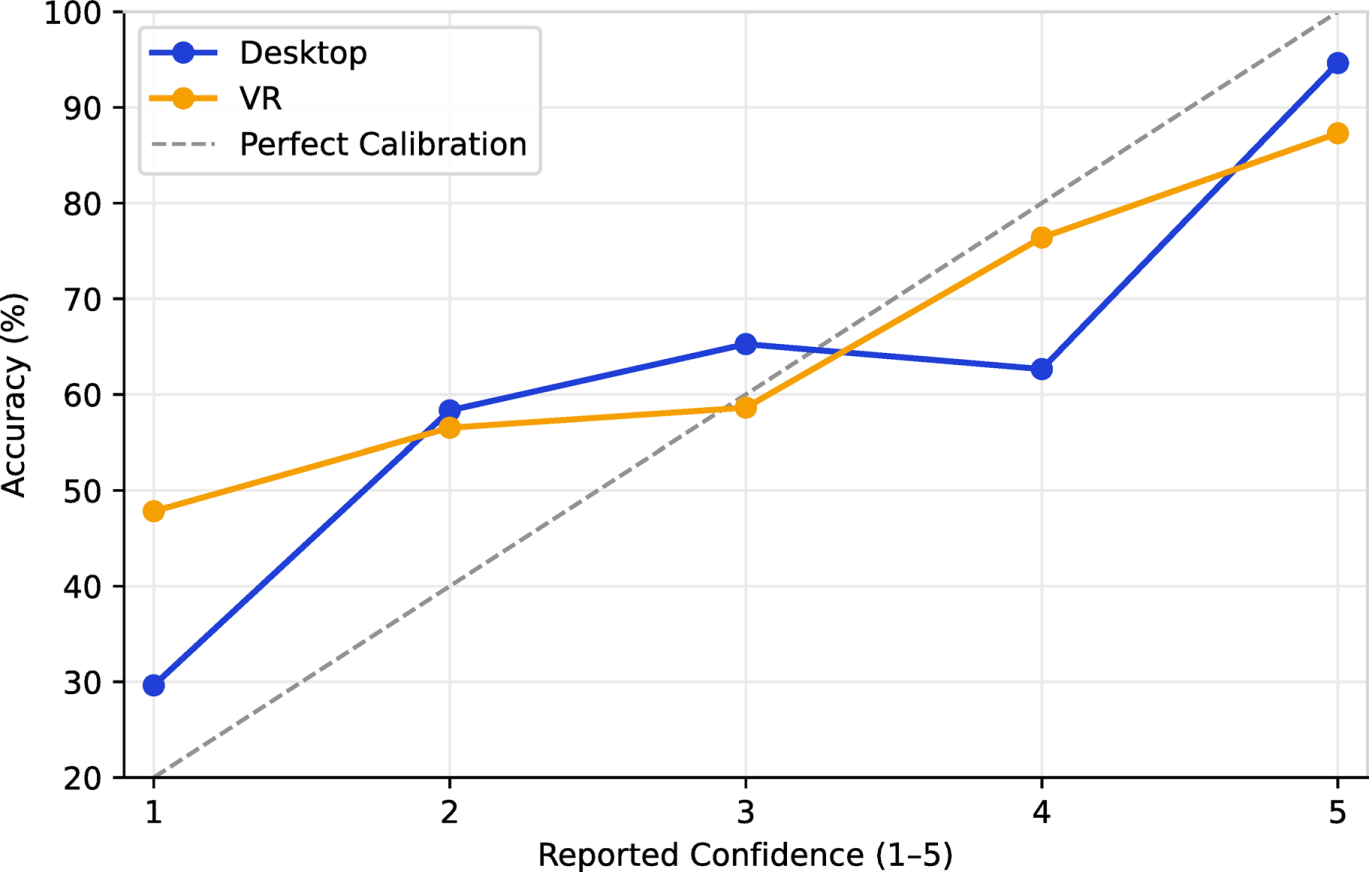
Confidence calibration for the two binary-scored items by modality. Accuracy is plotted against reported confidence levels for DI and VR conditions. The dashed line indicates perfect calibration. VR virtual reality.

In an exploratory analysis, confidence was examined for the two binary-scored items per model, namely liver-segment identification and vessel-pathway tracing, since accuracy–confidence calibration requires a binary correct/incorrect outcome. Out of 692 task responses, 665 (96.1%) included confidence ratings. Descriptively, the proportion of high-confidence responses (Likert 4–5) was slightly higher in VR (45.9%) than in DI (41.7%), and mean confidence was numerically higher in VR (3.30 ± 1.20 vs. 3.21 ± 1.19). However, this difference did not reach statistical significance (Mann–Whitney U = 52,645.5, *p* = 0.305) (Fig. 8). Accuracy rose with reported confidence in both conditions (Fig. 9). The confidence-accuracy association was comparable across modalities (VR: γ = 0.41, 95% CI 0.26–0.55; DI: γ = 0.41, 95% CI 0.27–0.55), with no evidence of superior calibration in either modality.

## Discussion

 This study examined how task complexity influences spatial-anatomical understanding in virtual reality compared with a conventional 2D desktop interface during preoperative liver surgery planning. Participants demonstrated significantly higher performance when evaluating complex models in the VR environment, while the performance difference between modalities was lower for simpler tasks. These findings suggest that VR offers advantages particularly for tasks involving higher spatial and cognitive demands. The superior performance observed in the complex models in VR can likely be attributed to several factors. From a cognitive load perspective, immersive visualization may reduce the mental effort required to reconstruct three-dimensional anatomy from two-dimensional projections, allowing users to devote more cognitive resources to meaningful spatial reasoning^45^. The immersive VR environment provides stereoscopic depth cues and allows users to engage their proprioceptive senses, thereby enhancing three-dimensional perception of complex models, whereas the absence of these cues lowers the overall level of spatial perception^46,47^. The immersive nature of VR also minimizes external distractions and may promote greater attentional focus during the evaluation process^48^. The absence of a clear VR advantage in the simpler tasks aligns with findings from both medical and engineering domains^18,36,46^. Prior research in the medical domain has suggested that the relative benefit of immersive visualization depends on task complexity, but direct empirical confirmation has been limited^18,36^. In the engineering context, Horvat et al. showed that performance differences between the two modalities were more evident when participants evaluated 3D computer-aided models of technical systems with higher structural complexity^46^. 

 The findings of this study provide further evidence for this pattern by demonstrating that VR facilitates higher spatial understanding specifically in tasks requiring simultaneous spatial reasoning about multiple lesions, while performance converges between modalities when only a single lesion must be evaluated. This result aligns with the observations of Rashidian et al. and Timonen et al., who reported superior outcomes in VR for intricate surgical planning and measurement tasks but negligible differences for simpler spatial identification exercises, such as the identification of anatomical landmarks^18,36^. Similarly, Hattab et al. found no performance difference between VR and DI conditions when participants assessed single-lesion liver models and surrounding liver vessels^39^. They suggested that, although immersion may aid spatial memory through egocentric cues, it did not improve recall of internal anatomical structures in their setting^39^. Building on this, the present study shows that when tasks involve greater cognitive complexity, such as the presence of multiple lesions, VR’s immersive properties confer a clear advantage in spatial understanding. Collectively, these findings indicate that the benefits of VR emerge most prominently under conditions of high task complexity and cognitive load. 

 Another explanation for the VR performance within the easy task was the participants’ prior familiarity with desktop-based interactions. Most participants were already familiar with operating a DI monitor using a mouse and keyboard, whereas the VR environment, with its headset and hand controllers, was a novel experience for the majority. Despite a brief introduction to the VR setup, adapting to a new interface and interaction style likely required additional time and practice. It is noteworthy that the easier models were presented at the beginning of the experiment, when participants were still becoming accustomed to the VR environment. Consequently, early evaluations may not fully reflect the potential performance achievable once users became more proficient. As the experiment progressed, particularly during the assessment of more complex models, participants appeared to use VR functionalities more effectively, suggesting a steep learning curve associated with the technology. 

 Regarding secondary outcomes examining the relationship between participants’ spatial ability, measured by the MRT and their task performance, the results showed that, for the complex models, higher MRT scores were associated with greater performance differences between the two modalities, favoring the VR condition. This directly contradicts the directional hypothesis we derived from Jang et al., who reported that learners with lower spatial ability benefit more from active manipulation in immersive environments; on that basis we had expected lower-MRT participants to gain most from VR. Instead, the advantage was concentrated among higher-MRT participants^47^. A plausible explanation is that participants with stronger intrinsic spatial skills adapted more quickly to the unfamiliar VR interface and exploited its features within the limited exploration window, while lower-MRT participants required more time to acclimate. We note, however, that this is a learning-curve account rather than evidence for the perceptual mechanism invoked elsewhere in this discussion. 

 In an exploratory analysis of confidence, mean confidence was marginally higher in VR but not significantly so, and the association between confidence and accuracy was comparable across modalities, with no indication that immersion improved the alignment between self-assessment and performance. 

 This study possesses several methodological and conceptual strengths. The participant cohort, medical students who had completed at least two years of training, represented an ideal population for evaluating spatial understanding in surgical contexts, combining sufficient anatomical knowledge with limited operative experience, thereby minimizing confounding effects of prior surgical exposure. The sample size was relatively large for this research domain, strengthening the robustness and interpretability of the findings. Methodologically, the study employed a rigorous randomized comparative crossover design with counterbalanced modality order, ensuring that each participant experienced both visualization modalities across multiple distinct 3D liver models while mitigating learning effects. Importantly, the explicit stratification of tasks by anatomical complexity enabled a more nuanced assessment of visualization-dependent performance differences, distinguishing this work from prior studies. Furthermore, both software platforms were deliberately designed with minimal differences in functionality and user interface, allowing observed effects to be attributed to visualization modality rather than software complexity. Finally, the inclusion of individual spatial ability assessments enabled an exploratory analysis of their association with performance across visualization modalities, providing insight into which participants may be more likely to benefit from VR-based approaches. 

 Several limitations should be acknowledged. First, most participants had limited experience with VR technology prior to their enrollment. Although a standardized introduction was provided, a brief familiarization period may not have been sufficient for participants to fully leverage all VR features. Second, although modality order was counterbalanced, easy cases were consistently presented before complex cases across both randomization sequences. Exposure to VR therefore increased across the session and was confounded with task complexity. Cumulative VR exposure was thus confounded with task complexity, and the larger VR advantage on complex tasks could reflect either a genuine complexity effect or progressive adaptation to the VR interface. Third, as the study cohort consisted of medical students without surgical training, the findings are limited to spatial understanding and cannot be directly extrapolated to the creation of operative plans or intraoperative decision-making. Finally, the design does not isolate a single mechanism underlying the VR advantage: the two conditions differed not only in stereoscopic depth and motion parallax but also in interaction mode, since x/z translation was disabled in VR to encourage embodied navigation whereas the desktop condition permitted full mouse and keyboard manipulation. The observed advantage therefore reflects the combined contribution of stereoscopic viewing, motion parallax, and embodied interaction, which the present design cannot separate. 

 Future studies should examine how long-term integration of VR within the clinical workflow affects spatial reasoning, learning curves and planning accuracy over time, ideally through longitudinal designs spanning medical students, surgical residents and practicing surgeons. Whether the benefits observed here generalize to clinically complex anatomy, characterized by vascular variants, infiltrative lesions or post-treatment changes, remains a hypothesis for future investigation under realistic anatomical conditions. 

 Although VR has often been viewed as a technological novelty, the present findings suggest that its advantages extend beyond visual immersion alone, providing measurable gains in spatial perception during complex tasks and enhancing spatial reasoning when cognitive demands are high. These effects appear to be more closely associated with task complexity than with organ-specific factors, suggesting potential relevance for other surgical and medical domains in which tasks require simultaneous spatial reasoning about multiple anatomical targets. Because the immersive condition combined stereoscopic depth, motion parallax and embodied navigation, these gains cannot be attributed to any single mechanism. By quantifying these benefits under controlled conditions, this study supports the integration of immersive VR into surgical education and preoperative planning, where it can serve as a practical tool to improve anatomical understanding and clinical decision-making.

## Supplementary Information

Below is the link to the electronic supplementary material.

Supplementary Material 1

Supplementary Material 2

## Data Availability

All data for this study is available in a fully anonymized format upon request from the corresponding author.
