# Supplementary material for: Effect of virtual reality on spatial–anatomical understanding in preoperative liver surgery: a randomized crossover study
Source: Sci Rep. 2026 Jul 8;16:21191. doi: 10.1038/s41598-026-61007-6 (PMC13347006; doi:10.1038/s41598-026-61007-6)
Supplement: Supplementary file 1 — Supplementary Material 1 [file 41598_2026_61007_MOESM1_ESM.pdf]

Explore MCQ:

1. In which liver lobe is the lesion located? (Please circle all that apply, multiple answers are possible)

    ◊ Left (Green lesion)
    ◊ Right (Yellow lesion)

Skip to question 2, if you decided for the left lobe
Skip to question 8, if you decided for the right lobe

The answer should refer to the part of the vessel closest to the lesion!!

Lesion in the left lobe:

2. Where is the lesion in relation to the left hepatic vein? (Please circle **all** that apply)

    ◊ medial / lateral / neither
    ◊ cranial / caudal / neither
    ◊ dorsal / ventral / neither
    ◊ Directly in contact with the structure / Not in contact with the structure

Confidence scale:

Not confident at all	Slightly confident	Somewhat confident	Fairly confident	Completely confident
1	2	3	4	5

3. Where is the lesion in relation to the middle hepatic vein? (Please circle **all** that apply)

    ◊ medial / lateral / neither
    ◊ cranial / caudal / neither
    ◊ dorsal / ventral / neither
    ◊ Directly in contact with the structure / Not in contact with the structure

Confidence scale:

Not confident at all	Slightly confident	Somewhat confident	Fairly confident	Completely confident
1	2	3	4	5

4. Where is the lesion in relation to the left hepatic artery? (Please circle **all** that apply)

◊ medial / lateral / neither
◊ cranial / caudal / neither
◊ dorsal / ventral / neither
◊ Directly in contact with the structure / Not in contact with the structure

Confidence scale:

Not confident at all	Slightly confident	Somewhat confident	Fairly confident	Completely confident
1	2	3	4	5

5. Where is the lesion in relation to the left portal vein branch? (Please circle **all** that apply)

◊ medial / lateral / neither
◊ cranial / caudal / neither
◊ dorsal / ventral / neither
◊ Directly in contact with the structure / Not in contact with the structure

Confidence scale:

Not confident at all	Slightly confident	Somewhat confident	Fairly confident	Completely confident
1	2	3	4	5

6. Identify the correct portal vein which was seen in the model. *(Please circle **one** that applies)*

◊ A

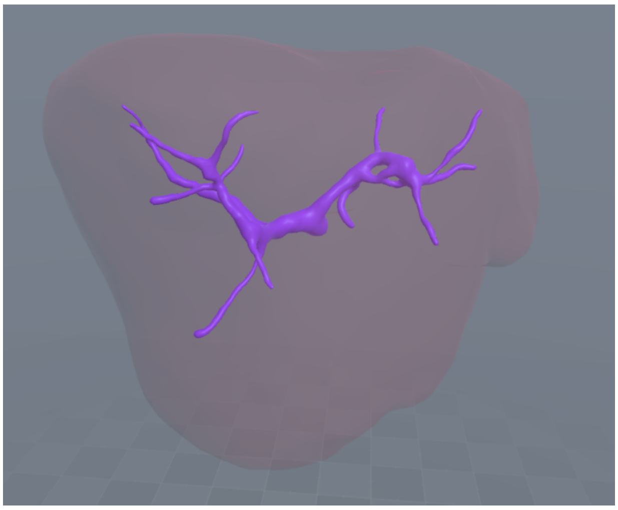

◊ B

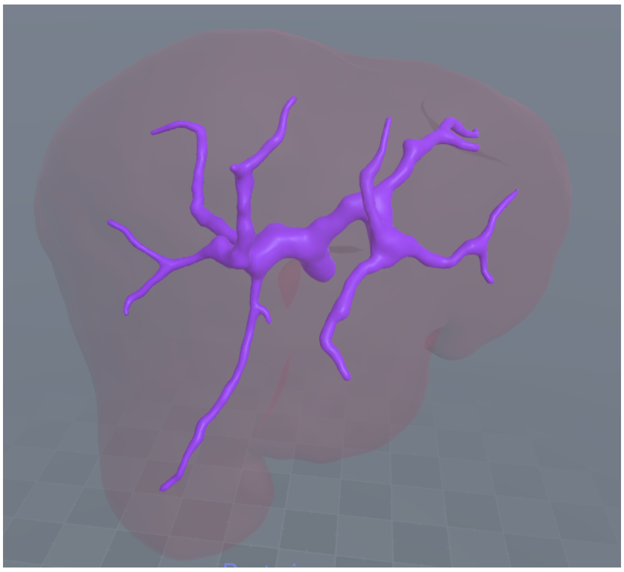

◊ C

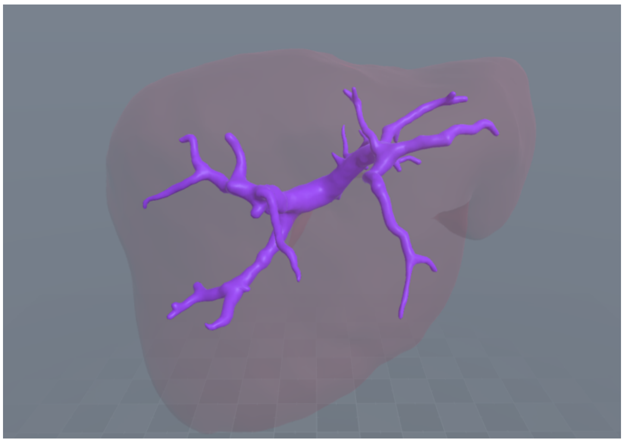

◊ D

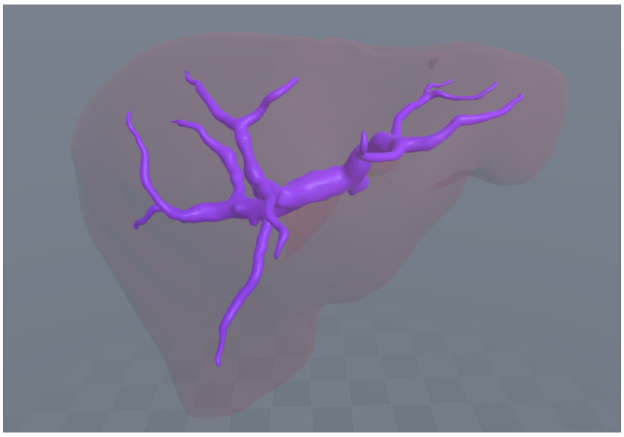

Confidence scale:

Not confident at all	Slightly confident	Somewhat confident	Fairly confident	Completely confident
1	2	3	4	5

7. In which Liver segment is the lesion located? (Choose the segment where the **largest mass** of the lesion is located)
   ◊ 1
   ◊ 2
   ◊ 3
   ◊ 4a
   ◊ 4b
   ◊ 5
   ◊ 6
   ◊ 7
   ◊ 8

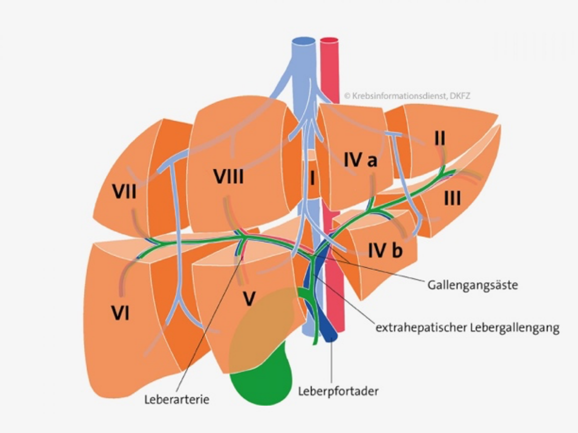

Figure 1

Confidence scale:

Not confident at all	Slightly confident	Somewhat confident	Fairly confident	Completely confident
1	2	3	4	5

Lesion in the right side of the liver

8. Where is the lesion in relation to the right hepatic vein? (Please circle **all** that apply)

   ◊ medial / lateral / neither
   ◊ cranial / caudal / neither
   ◊ dorsal / ventral / neither
   ◊ Directly in contact with the structure / Not in contact with the structure

Confidence scale:

Not confident at all	Slightly confident	Somewhat confident	Fairly confident	Completely confident
1	2	3	4	5

9. Where is the lesion in relation to the middle hepatic vein? (Please circle **all** that apply)
   - ◊ medial / lateral / neither
   - ◊ cranial / caudal / neither
   - ◊ dorsal / ventral / neither
   - ◊ Directly in contact with the structure / Not in contact with the structure

Confidence scale:

Not confident at all	Slightly confident	Somewhat confident	Fairly confident	Completely confident
1	2	3	4	5

10. Where is the lesion in relation to the right hepatic artery? (Please circle **all** that apply)
    - ◊ medial / lateral / neither
    - ◊ cranial / caudal / neither
    - ◊ dorsal / ventral / neither
    - ◊ Directly in contact with the structure / Not in contact with the structure

Confidence scale:

Not confident at all	Slightly confident	Somewhat confident	Fairly confident	Completely confident
1	2	3	4	5

11. Where is the lesion in relation to the right portal vein branch? (Please circle **all** that apply)
    - ◊ medial / lateral / neither
    - ◊ cranial / caudal / neither
    - ◊ dorsal / ventral / neither
    - ◊ Directly in contact with the structure / Not in contact with the structure

Confidence scale:

Not confident at all	Slightly confident	Somewhat confident	Fairly confident	Completely confident
1	2	3	4	5

12. Identify the correct liver vein which was seen in the model. *(Please circle **one** that applies)*

◊ A

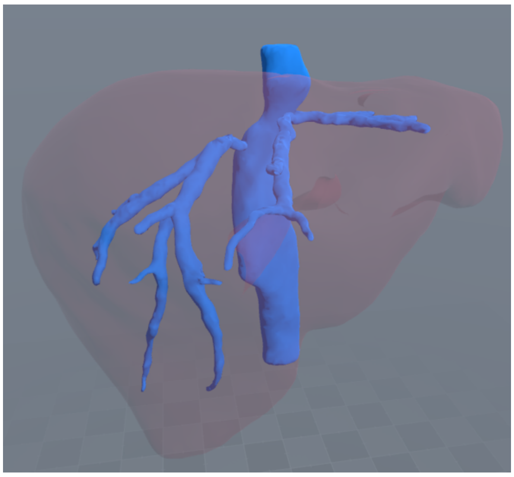

◊ B

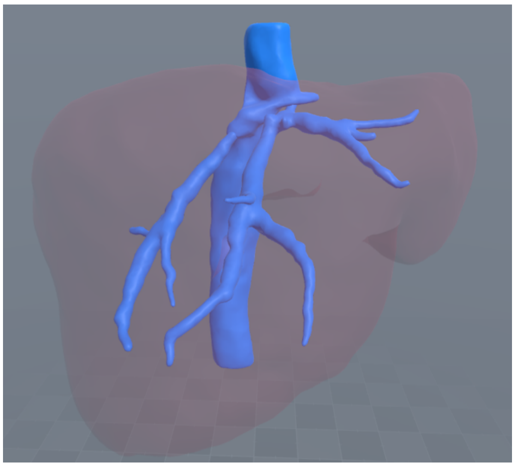

◊ C

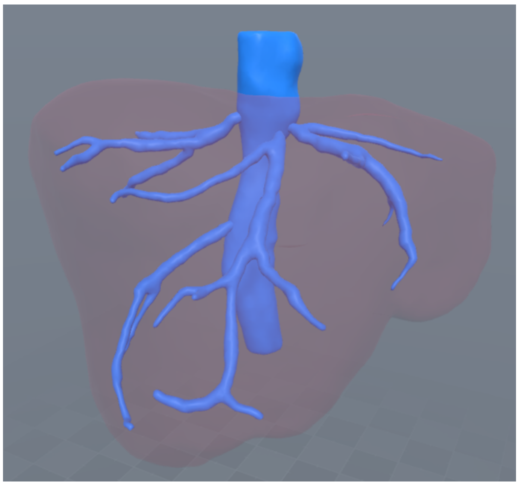

◊ D

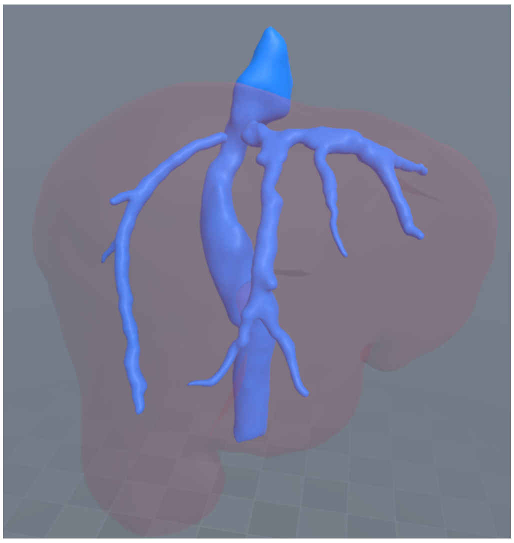

Confidence scale:

Not confident at all	Slightly confident	Somewhat confident	Fairly confident	Completely confident
1	2	3	4	5

13. In which Liver segment is the lesion located? (Choose the segment where the **largest mass** of the lesion is located)

◊ 1
◊ 2
◊ 3
◊ 4a
◊ 4b
◊ 5
◊ 6
◊ 7
◊ 8

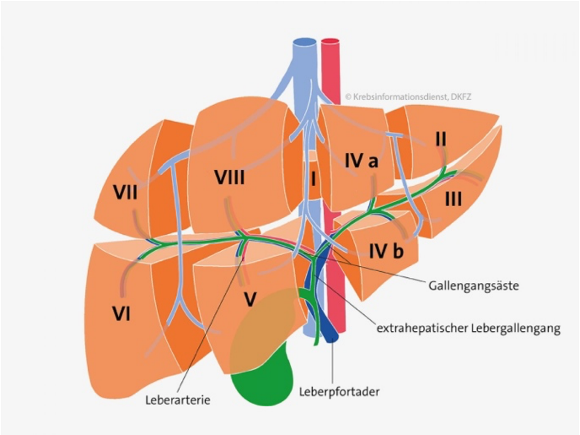

Figure 1

Confidence scale:

Not confident at all	Slightly confident	Somewhat confident	Fairly confident	Completely confident
1	2	3	4	5

Figures:

1. https://www.krebsinformationsdienst.de/tumorarten/metastasen/lebermetastasen/grundlagen.php - Zuletzt geöffnet: 18.03.24 , Asena Tunali © Krebsinformationsdienst, DKFZ
