# Supplementary material for: Effect of virtual reality on spatial–anatomical understanding in preoperative liver surgery: a randomized crossover study
Source: Sci Rep. 2026 Jul 8;16:21191. doi: 10.1038/s41598-026-61007-6 (PMC13347006; doi:10.1038/s41598-026-61007-6)
Supplement: Supplementary file 2 — Supplementary Material 2 [file 41598_2026_61007_MOESM2_ESM.pdf]

Consent to Publish Identifiable Images

Scientific Reports (Nature Portfolio)

Manuscript title:

Effect of virtual reality on spatial–anatomical understanding in preoperative liver surgery: a randomized crossover study

Corresponding authors: Anton Zolkin and Igor M. Sauer, Department of Surgery, Experimental Surgery, Charité – Universitätsmedizin Berlin.

Statement of consent

I, the undersigned, confirm that I am the individual whose face and/or other identifiable features appear in Figures 3 and 4 of the above manuscript.

I have read the manuscript, or the relevant figure has been shown and explained to me, and I understand the context in which my image will be used.

I give my consent for this image, together with any associated text, to be published in Scientific Reports (Nature Portfolio). I understand that the article will be published under an open-access licence, that the image will be freely available on the internet and that it may be seen by the general public worldwide. I understand that the image may also be used in derivative works, indexed by third parties and that it cannot be fully withdrawn once published.

I confirm that this consent is given freely and that I have had the opportunity to ask questions about the use of my image.

Signature

Full name: Anton Zolkin

Signature:

Date: 23.06.2026

Place: Berlin, Germany
